# Tópicos Emergentes em Insuficiência Cardíaca: Perspectivas Futuras

**DOI:** 10.36660/abc.20201205

**Published:** 2020-12-01

**Authors:** Mucio Tavares de Oliveira, Humberto Villacorta, Marcelo Imbroinise Bittencourt, Antônio Carlos Pereira Barretto, Evandro Tinoco Mesquita, Luis Eduardo Rohde

**Affiliations:** 1 Instituto do Coração Hospital das Clínicas Universidade de São Paulo São PauloSP Brasil Instituto do Coração do Hospital das Clínicas da Universidade de São Paulo,São Paulo, SP - Brasil; 2 Faculdade de Medicina Universidade Federal Fluminense NiteróiRJ Brasil Faculdade de Medicina - Universidade Federal Fluminense, Niterói, RJ - Brasil; 3 Clínica de Insuficiência Cardíaca e Cardiomiopatias Hospital Universitário Pedro Ernesto Universidade do Estado do Rio de Janeiro Rio de JaneiroRJ Brasil Clínica de Insuficiência Cardíaca e Cardiomiopatias - Hospital Universitário Pedro Ernesto - Universidade do Estado do Rio de Janeiro, Rio de Janeiro, RJ - Brasil; 4 Faculdade de Medicina Universidade de São Paulo Serviço de Prevenção e Reabilitação São PauloSP Brasil Faculdade de Medicina da Universidade de São Paulo - Serviço de Prevenção e Reabilitação do Instituto do Coração, São Paulo, SP - Brasil; 5 Grupo de Insuficiência Cardíaca Avançada Serviço de Cardiologia Hospital de Clínicas de Porto Alegre Porto AlegreRS Brasil Grupo de Insuficiência Cardíaca Avançada do Serviço de Cardiologia do Hospital de Clínicas de Porto Alegre, Porto Alegre, RS - Brasil; 6 Faculdade de Medicina Universidade Federal do Rio Grande do Sul Porto AlegreRS Brasil Faculdade de Medicina da Universidade Federal do Rio Grande do Sul, Porto Alegre, RS - Brasil

**Keywords:** Biomarcadores, Avaliação Genética, Omecamtiv Mecarbil, Mavacamten, Telemedicina, Imunização, Vacinas, Danon, Fabry, Amiloidose

## Introdução

O *Heart Failure Summit 2020* abordou novas perspectivas de avaliação, diagnóstico, estratificação de risco e tratamento da insuficiência cardíaca (IC), as quais em breve poderão estar disponíveis para ampla aplicabilidade clínica. Essas estratégias envolvem o uso de novos biomarcadores, a avaliação genética, novos potenciais alvos terapêuticos e a medicina personalizada.

### Novos Biomarcadores

Entre os novos biomarcadores, citamos dois relacionados ao processo de fibrose. A galectina-3 (Gal-3) é expressa em vários tecidos e células, incluindo o miocárdio. No coração, ela ativa fibroblastos quiescentes, transformando-os em miofibroblastos que alteram a matriz extracelular, produzindo fibrose, sendo preditora de remodelamento na IC. Além de ser um marcador de risco, a Gal-3 parece participar ativamente do processo de fibrose, sendo um potencial alvo terapêutico.^1^ O ST2 solúvel é outro marcador de fibrose na IC, fornecendo informações adicionais aos peptídeos natriuréticos e troponinas.^2^ Mais recentemente, o fator de diferenciação de crescimento-15 (GDF-15) tem-se mostrado preditor de eventos na IC.^3^

Embora esses biomarcadores sejam promissores, ainda não temos um grande estudo mostrando que adicionam informação ao manuseio convencional.

### Avaliação Genética

Os avanços na genética estão melhorando a compreensão das diversas cardiopatias hereditárias, em especial as cardiomiopatias, causas frequentes de IC. Doenças como cardiomiopatia dilatada e cardiomiopatia hipertrófica têm sido impactadas nesse sentido.^4^ Evidências nessa área vêm esclarecendo aspectos etiopatogênicos, como variantes em truncamento de genes. Outro cenário que a genética ajudou a mudar nossa forma de avaliação foi a cardiomiopatia arritmogênica, tema de consenso recentemente publicado.^5^ Antigamente associada exclusivamente a cardiomiopatia do ventrículo direito determinada por mutações desmossomais, hoje sabemos que abrange um amplo espectro de doenças genéticas, sistêmicas e inflamatórias. A incorporação do sequenciamento de última geração vem aumentando a sensibilidade dos testes genéticos, o que possibilita diagnóstico precoce com perspectiva de intervenção. Entretanto, nem todos estão cientes da aplicabilidade dessa ferramenta, assim como das armadilhas que surgem com a sua incorporação. Fica clara a necessidade de buscar formas mais eficientes da utilização da genética, especialmente no aconselhamento familiar, trazendo resultados seguros e sustentáveis na gestão do cuidado desses pacientes e suas famílias.

### Novos Alvos Terapêuticos

A miosina como alvo de tratamento nos traz duas novas drogas com resultados promissores nos estudos iniciais de fase 1 e 2 no tratamento da IC e da cardiomiopatia hipertrófica.

#### Omecamtiv mecarbil

É um ativador seletivo da miosina, e essa ativação trataria a alteração central da disfunção ventricular, melhorando a contração ventricular comprometida nos casos de IC com fração de ejeção reduzida. Seu mecanismo de ação é diferente dos tratamentos atuais que bloqueiam a elevada estimulação neuro-hormonal. Os estudos mecanísticos, como o ATOMIC-AHF ( *Acute Treatment with Omecamtiv Mecarbil to Increase Contractility in Acute Heart Failure* ) e o COSMIC-HF ( *Chronic Oral Study of Myosin Activation to Increase Contractility in Heart Failure* ), mostraram que a droga melhora a contratilidade, melhorando a fração de ejeção, o volume ejetado e o débito cardíaco, além de outros parâmetros que indicam melhora da função cardíaca. Os estudos mostraram que promove redução dos níveis de NT-proBNP. Identificou-se também elevação dos níveis de troponina, sem alterações clínicas nos estudos realizados. O estudo ATOMIC-AHF, entretanto, em pacientes com IC aguda, não documentou redução da dispneia nos pacientes tratados. O estudo GALACTIC-HF (Registrational Study With Omecamtiv Mecarbil/AMG 423 to Treat Chronic Heart Failure With Reduced Ejection Fraction) não demonstrou benefício da droga na mortalidade a longo prazo e uma redução de apenas 5% para primeira hospitalização.^6^ Estudos em populações com IC aguda e com IC terminal são necessários para definir a utilidade da droga.

#### Mavacamten

O mavacamten é um inibidor específico da miosina, e estudos vêm mostrando que a sua prescrição promove melhora no desempenho físico dos portadores de cardiomiopatia hipertrófica obstrutiva, com melhora do consumo de oxigênio.^7^ Com sua prescrição, documentou-se redução dos níveis de NT-proBNP e de troponina, sugerindo redução do estresse na parede miocárdica, redução da obstrução ventricular e redução da fração de ejeção. No estudo MAVERICK, os eventos colaterais foram frequentes e um percentual não desprezível de pacientes apresentou redução exagerada da fração de ejeção, reversível com a suspensão da droga. O acerto da dose parece ser fundamental e se propõe que seja avaliado com base na resposta da fração de ejeção. Precisamos aguardar os estudos de sobrevida para ter certeza de que a droga é segura e entender melhor como avaliar a redução da contratilidade e da fração de ejeção com o medicamento.

## Telemedicina

A telemedicina é uma ampla plataforma de estratégias singulares com complexidades variadas que surgiram na área médica como uma ferramenta promissora para melhorar a adesão de pacientes com doenças crônicas e reduzir as desigualdades em saúde. Infelizmente, a aplicabilidade da telemedicina tem sido errática na América Latina e em muitas partes do mundo, devido às dificuldades logísticas para estabelecer estruturas tecnológicas de suporte que sejam eficientes e viáveis para muitos pacientes. Além disso, várias estratégias de telemonitoramento foram testadas em IC com resultados inconsistentes. A falta de eficácia de forma conclusiva pode ser explicada por diferenças no conteúdo e intensidade de cada intervenção e pela natureza heterogênea das populações de pacientes incluídos em diferentes estudos. A maioria dos estudos que demonstram os efeitos benéficos das estratégias de telemedicina na IC envolveu pacientes instáveis e vulneráveis com uma hospitalização recente. Também parece essencial que estratégias específicas sejam adaptadas para cada ambiente de saúde, considerando aspectos sociais, econômicos, cognitivos e culturais. Uma análise de 15 revisões sistemáticas sobre a eficácia dessas estratégias para pacientes com IC sugere que as intervenções de telemonitoramento domiciliar reduzem o risco relativo de mortalidade por todas as causas e hospitalizações relacionadas à IC em comparação com o tratamento usual. As reduções de risco na mortalidade e hospitalizações por todas as causas pareceram ser maiores em pacientes que receberam alta recentemente (≤ 28 dias) em cenários de descompensações agudos após uma exacerbação recente de IC.^8^

## Novas Terapias na Insuficiência Cardíaca com Fração de Ejeção Preservada (ICFEP)

Entre as terapias para ICFEp, as que mais têm chamado a atenção e curiosidade são os inibidores do cotransportador de sódio-glicose (iSGLT2), como a empaglifozina [EMPEROR-Preserved ( *Empagliflozin outcome trial in patients with chronic heart failure with preserved ejection fraction* ) e EMBRACE-HF ( *Empagliflozin impact on hemodynamics in patients with heart failure* )], a dapaglifozina [PRESERVED-HF ( *Dapagliflozin in preserved ejection fraction heart failure* )] e a ertuglifozina [ERADICATE-HF ( *Ertugliflozin trial in diabetes with preserved or reduced ejection fraction mechanistic evaluation in heart failure* )]. Outras drogas também estão em fase de avaliação, como TRC4186 (inibidor dos produtos finais de glicolização avançada), trimetazidina, praliciguat e vericiguat (estimulante da guanilato ciclase solúvel), neladenoson (agonista parcial do receptor A1 da adenosina) e o pirfenidone (agente antifibrótico), como também o procedimento de abertura de uma comunicação interatrial por cateter a fim de aliviar a pressão elevada do átrio esquerdo.

## Imunizações na IC

Até pouco tempo, não havia dados sobre o impacto da *influenza* sobre os desfechos em pacientes com IC,^9^ mas ficou demonstrado em estudo populacional a relação entre a estação de *influenza* e a ocorrência de maior número de internações por IC, coincidindo em quatro períodos consecutivos. Em uma subanálise do estudo PARADIGM (P *rospective comparison of ARNI with ACEI to determine impact on global mortality and morbidity* ), 21% dos participantes receberam vacinação contra *influenza* e nestes houve uma redução de morte total de 19% após ajuste de propensão. Em um estudo de coorte dinamarquês com 134.048 pacientes com IC, uma ou mais vacinações entre 2003 e 2015 resultaram igualmente em redução de 18% na mortalidade total e mortalidade por todas as causas e mais de três vacinações, a uma redução de 28% na mortalidade total e de 29% na mortalidade cardiovascular.^10^ Um estudo com banco de dados de 6.435 pacientes com IC, sendo 695 vacinados antes ou durante o inverno de 2017/2018, houve redução de 22% na morte total e 17% em morte cardiovascular ou internação por IC, sendo o benefício da vacinação sobre a morte total maior em pacientes com mais de 70 anos, com redução de mais de 25%.^11^ Não há estudos sobre o impacto da vacinação para pneumococo. Diversos estudos prospectivos estão em fase de inclusão de pacientes.

## IC Personalizada

A promessa da medicina personalizada na IC vai progressivamente se tornando uma nova realidade e um novo campo apoiado na cardioimagem, na genômica e nas ciências de dados. Na presente década, ocorreu um importante desafio do governo norte-americano, apoiando iniciativas de pesquisa para doenças crônicas visando incorporar o paradigma da medicina de precisão, e esse esforço tem apresentado importantes avanços em diferentes centros acadêmicos. A ICFEp é hoje um dos maiores desafios na prática clínica pela ausência de medicamentos capazes de reduzir a morbimortalidade. A ICFEp vem sendo estudada com um novo olhar, apoiando-se nas técnicas de bioinformática, identificando redes fenotípicas e buscando identificar padrões que possam cursar com diferentes prognósticos e, possivelmente, apresentar respostas diferentes frente aos medicamentos.^12^

Apoiando-se nas técnicas de bioinformática e de cientistas de dados, tem sido possível predizer a evolução clínica das cardiomiopatias hereditárias, como as doenças de Danon e Fabry.^13^ A miocardiopatia hipertrófica tem apresentado grandes avanços com a possibilidade de caracterizar fenocópias – condições que simulam a apresentação morfológica da miocardiopatia hipertrófica, tais como a doença de Fabry, amiloidose cardíaca, doença de Pompe, doença de Danon, entre outras. As técnicas que manipulam o código genético, como o sistema de repetições palindrômicas curtas agrupadas e regularmente interespaçadas (CRISPR), têm o potencial futuro de tratarem doenças monogênicas e estão sendo empregadas em modelos animais.

O maior avanço da presente década tem sido a realização do diagnóstico da amiloidose cardíaca e da caracterização de seus principais tipos, combinando imagem molecular, biomarcadores e testes genéticos.^14^Há algoritmos que empregam dosagem sérica das cadeias leves de imunoglobulina e imunofixação no sangue e urina, cintilografia cardíaca com pirofosfato de tecnécio em associação com abordagem multimodal com técnicas avançadas de ecodopplercardiografia com *speckle tracking* e da ressonância cardíaca em combinação com teste genético. Essa abordagem tem permitido a substituição da biópsia de tecido gorduroso e/ou cardíaco para correta identificação de amiloidose decorrente da mutação da transtirretina. Os novos medicamentos, como o tafamidis, e aqueles que interferem com a transcrição do RNA, como o patisiran, são exemplos da personalização do tratamento. Um recente relato de caso mostra a regressão e normalização da cintilografia miocárdica com pirofosfato de tecnécio no paciente tratado com esses novos medicamentos no curso da amiloidose cardíaca por uma mutação da TTR, representando esse novo futuro.^15^

## Lista de Participantes do Heart Failure Summit Brazil 2020 / Departamento de Insuficiência Cardíaca - DEIC/SBC

Aguinaldo Freitas Junior, Andréia Biolo, Antonio Carlos Pereira Barretto, Antônio Lagoeiro Jorge, Bruno Biselli, Carlos Eduardo Montenegro, Denilson Campos de Albuquerque, Dirceu Rodrigues de Almeida, Edimar Alcides Bocchi, Edval Gomes dos Santos Júnior, Estêvão Lanna Figueiredo, Evandro Tinoco Mesquita, Fabiana G. Marcondes-Braga, Fábio Fernandes, Fabio Serra Silveira, Felix José Alvarez Ramires, Fernando Atik, Fernando Bacal, Flávio de Souza Brito, Germano Emilio Conceição Souza, Gustavo Calado de Aguiar Ribeiro, Humberto Villacorta Jr., Jefferson Luis Vieira, João David de Souza Neto, João Manoel Rossi Neto, José Albuquerque de Figueiredo Neto, Lídia Ana Zytynski Moura, Livia Adams Goldraich, Luís Beck-da- Silva, Luís Eduardo Paim Rohde, Luiz Claudio Danzmann, Manoel Fernandes Canesin, Marcelo Bittencourt, Marcelo Westerlund Montera, Marcely Gimenes Bonatto, Marcus Vinicius Simões, Maria da Consolação Vieira Moreira, Miguel Morita Fernandes da Silva, Monica Samuel Avila, Mucio Tavares de Oliveira Junior, Nadine Clausell, Odilson Marcos Silvestre, Otavio Rizzi Coelho Filho, Pedro Vellosa Schwartzmann, Reinaldo Bulgarelli Bestetti, Ricardo Mourilhe Rocha, Sabrina Bernadez Pereira, Salvador Rassi, Sandrigo Mangini, Silvia Marinho Martins, Silvia Moreira Ayub Ferreira, Victor Sarli Issa.
