# Supplementary material for: Tópicos Emergentes em Insuficiência Cardíaca: Perspectivas Futuras
Source: Arq Bras Cardiol. 2020 Dec 1;115(6):1197–200. [Article in Portuguese] doi: 10.36660/abc.20201205 (PMC8133736; doi:10.36660/abc.20201205)
Supplement: Translation

# Translation: Emerging Topics in Heart Failure: Future Perspectives

**Authors:** Mucio Tavares de Oliveira, Humberto Villacorta, Marcelo Imbroinise Bittencourt, Antônio Carlos Pereira Barretto, Evandro Tinoco Mesquita, Luis Eduardo Rohde

**Affiliations:** 1 Brasil Instituto do Coração do Hospital das Clínicas da Universidade de São Paulo, São Paulo, SP - Brasil; 2 Brasil Faculdade de Medicina - Universidade Federal Fluminense, Niterói, RJ - Brasil; 3 Brasil Clínica de Insuficiência Cardíaca e Cardiomiopatias - Hospital Universitário Pedro Ernesto - Universidade do Estado do Rio de Janeiro, Rio de Janeiro, RJ - Brasil; 4 Brasil Faculdade de Medicina da Universidade de São Paulo - Serviço de Prevenção e Reabilitação do Instituto do Coração, São Paulo, SP - Brasil; 5 Brasil Grupo de Insuficiência Cardíaca Avançada do Serviço de Cardiologia do Hospital de Clínicas de Porto Alegre, Porto Alegre, RS - Brasil; 6 Brasil Faculdade de Medicina da Universidade Federal do Rio Grande do Sul, Porto Alegre, RS - Brasil

**Keywords:** Biomarkers, Genetic Assessment, Omecamtiv Mecarbil, Mavacamten, Telemedicine, Imunization, Vaccins, Danon, Fabry, Amiloidosis

## Introduction

The *Heart Failure Summit Brazil 2020* addressed new perspectives for assessment, diagnosis, risk stratification, and management of heart failure (HF), which may soon be available for widespread use in the clinic. These strategies involve the use of novel biomarkers, genetic assessment, potential new therapeutic targets, and personalized medicine.

### Novel Biomarkers

Among novel biomarkers, two—both related to the process of fibrosis—are worthy of note. Galectin-3 (Gal-3) is expressed in various tissues and cells, including the myocardium. In the heart, Gal-3 activates quiescent fibroblasts, transforming them into myofibroblasts that alter the extracellular matrix to produce fibrosis; research has shown it is a predictor of remodeling in HF. In addition to being a marker of risk, Gal-3 appears to play an active role in the fibrosis process, and may thus constitute a potential therapeutic target.^1^Soluble ST2 is another marker of fibrosis in HF, providing additional information as an adjunct to natriuretic peptides and troponins.^2^More recently, growth differentiation factor-15 (GDF-15) has been shown to be a predictor of events in HF.^3^

Although these biomarkers are promising, there is still no conclusive evidence from large studies that they add information to conventional.

### Genetic Assessment

Advances in genetics are improving our understanding of the various hereditary heart diseases, especially cardiomyopathies, which are frequent causes of HF. Diseases such as dilated cardiomyopathy and hypertrophic cardiomyopathy have been impacted by such research;^4^new evidence has clarified certain etiopathogenetic aspects. One example is the finding that truncating variants of the titin gene account for up to a quarter of all dilated cardiomyopathies. Advances in genetics have also changed the approach to initial assessment of arrhythmogenic cardiomyopathy, the topic of a recently published consensus statement.^5^Once associated exclusively with right ventricular cardiomyopathy caused by mutations in desmosomal protein genes, we now know that this umbrella term covers a wide spectrum of genetic, systemic, and inflammatory conditions. The advent of next-generation sequencing has increased the sensitivity of genetic tests, which allows for early diagnosis with an interventional perspective. However, not all clinicians are aware of the applicability of this tool, nor of the pitfalls that arise with its incorporation into practice. There is a clear need for more efficient ways of using genetics, especially in family counseling, to achieve more safe and sustainable outcomes in the management of these patients and their families.

### New Therapeutic Targets

Two new drugs targeting myosin have yielded promising results in phase 1 and 2 trials for the treatment of HF and hypertrophic cardiomyopathy.

#### Omecamtiv Mecarbil

This is a selective myosin activator and is thus believed to address the core abnormality of ventricular dysfunction, improving compromised ventricular contraction in cases of HF with reduced ejection fraction. Its mechanism of action is distinct from that of current treatments, which counteract elevated neurohormonal stimulation. Mechanistic trials, such as ATOMIC-AHF (Acute Treatment with Omecamtiv Mecarbil to Increase Contractility in Acute Heart Failure) and COSMIC-HF (Chronic Oral Study of Myosin Activation to Increase Contractility in Heart Failure), have shown that this drug improves contractility, improving ejection fraction, stroke volume, and cardiac output, in addition to other parameters suggestive of improved cardiac function. Studies have shown that omecamtiv mecarbil promotes a reduction in NT-proBNP levels. Elevation of troponin levels, with no clinical repercussions, has also been reported. Nevertheless, the ATOMIC-AHF study of patients with acute HF did not observe a reduction in dyspnea in patients treated with the drug. A study, GALACTIC-HF (Registrational Study With Omecamtiv Mecarbil/AMG 423 to Treat Chronic Heart Failure With Reduced Ejection Fraction) fail to demonstrate long-term survival benefit, and showed only a 5% reduction for first HF hospitalization.^6^Further studies in acute HF patients and patients with terminal HF are necessary to stablish the usefulness of this drug.

#### Mavacamten

Mavacamten is a cardiac myosin-specific inhibitor. Studies have shown that it enhances physical performance in patients with obstructive hypertrophic cardiomyopathy, with improved oxygen consumption.^7^Its administration was associated with a reduction in NT-proBNP and troponin levels, suggesting reductions in stress in the myocardial wall, ventricular obstruction, and ejection fraction. In the MAVERICK study, adverse events were frequent and a not-insignificant percentage of patients experienced excessive decline in ejection fraction, which was however reversible upon discontinuation. Proper dose adjustment appears to be essential, and the ejection fraction response has been proposed as a guide. Again, survival studies are needed to ensure that mavacamten is safe and to better understand the reductions in contractility and ejection fraction that appear to follow its use.

## Telemedicine

Telemedicine is a broad platform of unique strategies with varied complexities that have emerged in the medical field as a promising tool to improve the treatment adherence of patients with chronic diseases and reduce health inequalities. Unfortunately, implementation of telemedicine has been erratic in Latin America and in many parts of the world, due to logistical difficulties in establishing the necessary technological support structures in a manner that is efficient and feasible for most patients. In addition, several telemonitoring strategies have been tested in HF, with inconsistent results. The lack of conclusive effectiveness may be explained by differences in the content and intensity of each intervention and by the heterogeneous nature of the patient populations included in different studies. Most studies that demonstrated beneficial effects of telemedicine strategies in HF have involved unstable and vulnerable patients with a history of recent hospitalization. It also seems essential that specific strategies be adapted to each health setting, considering social, economic, cognitive, and cultural aspects. An analysis of 15 systematic reviews on the effectiveness of these strategies for patients with HF suggests that home telemonitoring interventions reduce the relative risk of all-cause mortality and HF-related hospitalization compared to usual care. Risk reductions in all-cause mortality and hospitalization appeared to be greater in patients who were recently discharged (≤ 28 days) in the setting of acute decompensation after a recent exacerbation of HF.^8^

## New Therapies in Heart Failure with Preserved Ejection Fraction (HFPEF)

Among promising therapies for HFpEF, those that have drawn the most attention and interest are the sodium-glucose cotransporter inhibitors (SGLT2i), such as empagliflozin (evaluated in the EMPEROR-Preserved [Empagliflozin outcome trial in patients with chronic heart failure with preserved ejection fraction] and EMBRACE-HF [Empagliflozin impact on hemodynamics in patients with heart failure] trials), dapagliflozin (evaluated in PRESERVED-HF [Dapagliflozin in preserved ejection fraction heart failure]), and ertugliflozin (ERADICATE-HF [Ertugliflozin trial in diabetes with preserved or reduced and reduced fraction mechanistic evaluation in heart failure]). Other drugs are also at the investigational stage, such as TRC4186 (an inhibitor of advanced glycation end products), trimetazidine, praliciguat and vericiguat (soluble guanylate cyclase stimulants), neladenoson (a partial adenosine A1 receptor agonist), and pirfenidone (an antifibrotic agent), as is an invasive procedure (creation of an atrial septal defect to relieve high pressure in the left atrium).

## Immunizations in HF

Until recently, there was no data on the impact of influenza on outcomes in patients with HF,^9^but a population-based study has since demonstrated a relationship between influenza season and higher incidence of hospitalization for HF, which coincided in four consecutive periods. In a subanalysis of the PARADIGM (Prospective comparison of ARNI with ACEI to determine impact on global mortality and morbidity) trial, the 21% of participants who had received the influenza vaccine were found to have a 19% lower overall mortality after propensity matching. In a Danish cohort study of 134,048 patients with HF, one or more vaccinations between 2003 and 2015 were also associated with an 18% reduction in overall mortality and all-cause mortality; more than three vaccinations resulted in a 28% reduction in overall mortality and a 29% reduction in cardiovascular mortality.^10^A database study of 6,435 patients with HF, 695 of whom were vaccinated before or during the winter of 2017–2018, showed a 22% reduction in overall mortality and a 17% reduction in cardiovascular death or hospitalization due to HF. The benefit of vaccination on overall mortality was greatest in patients over age 70 years, with a >25% reduction.^11^There are no studies on the impact of pneumococcal vaccination. Several prospective studies are at the enrollment stage.

## Personalized HF Management

The promise of personalized medicine in HF management is progressively becoming reality and a new field in its own right, supported by cardiac imaging, genomics, and data sciences. In 2015, the U.S. government launched the Precision Medicine Initiative, a major challenge and research effort designed to support research initiatives for chronic diseases aimed at incorporating the precision medicine paradigm. This effort has led to significant advances at several academic centers. HFpEF is currently one of the greatest challenges in clinical practice due to the absence of drugs capable of reducing morbidity and mortality. Accordingly, a new outlook for HFpEF research has arisen which relies on bioinformatics techniques and identification of phenotypic networks, seeking to identify patterns that may be associated with different prognoses and, possibly, present differential responses to pharmacotherapy.^12^

Leveraging bioinformatics and data science techniques has enabled successful prediction of the clinical course of hereditary cardiomyopathies such as Danon disease and Fabry disease.^13^Great strides have been made in hypertrophic cardiomyopathy with the possibility of characterizing phenocopies—conditions that mimic the morphological presentation of hypertrophic cardiomyopathy, such as Fabry disease, cardiac amyloidosis, Pompe disease, Danon disease, etc. Genetic manipulation techniques, such as clustered regularly interspersed short palindromic repeats (CRISPR)-based gene editing, have potential for future utility in the treatment of monogenic diseases, and are already being tested in animal models.

For diagnosis of cardiac amyloidosis and characterization of its main subtypes through a combination of molecular imaging, biomarkers, and genetic testing have been used.^14^Serum measurement of immunoglobulin light chain and immunofixation in blood and urine, technetium pyrophosphate myocardial perfusion scanning, a multimodal approach of advanced Doppler echocardiography techniques such as speckle tracking, and cardiac magnetic resonance, all in combination with genetic tests. This approach has successfully replaced adipose tissue and/or endomyocardial biopsy for the accurate diagnosis of transthyretin-mediated cardiac amyloidosis (ATTR-CM). New drugs with disease-modifying capacity, such as the transthyretin stabilizer tafamidis and the small interfering RNA molecule patisiran, are examples of the increasingly personalized approach to management of cardiac amyloidosis. A recent case report of regression and normalization of technetium pyrophosphate myocardial perfusion scan findings in a patient with ATTR-CM treated with these new drugs is representative of this new and future paradigm.^15^

## List of participants of the Heart Failure Summit Brazil 2020 / Heart Failure Department - Brazilian Society of Cardiology

Aguinaldo Freitas Junior, Andréia Biolo, Antonio Carlos Pereira Barretto, Antônio Lagoeiro Jorge, Bruno Biselli, Carlos Eduardo Montenegro, Denilson Campos de Albuquerque, Dirceu Rodrigues de Almeida, Edimar Alcides Bocchi, Edval Gomes dos Santos Júnior, Estêvão Lanna Figueiredo, Evandro Tinoco Mesquita, Fabiana G. Marcondes-Braga, Fábio Fernandes, Fabio Serra Silveira, Felix José Alvarez Ramires, Fernando Atik, Fernando Bacal, Flávio de Souza Brito, Germano Emilio Conceição Souza, Gustavo Calado de Aguiar Ribeiro, Humberto Villacorta Jr., Jefferson Luis Vieira, João David de Souza Neto, João Manoel Rossi Neto, José Albuquerque de Figueiredo Neto, Lídia Ana Zytynski Moura, Livia Adams Goldraich, Luís Beck-da- Silva, Luís Eduardo Paim Rohde, Luiz Claudio Danzmann, Manoel Fernandes Canesin, Marcelo Bittencourt, Marcelo Westerlund Montera, Marcely Gimenes Bonatto, Marcus Vinicius Simões, Maria da Consolação Vieira Moreira, Miguel Morita Fernandes da Silva, Monica Samuel Avila, Mucio Tavares de Oliveira Junior, Nadine Clausell, Odilson Marcos Silvestre, Otavio Rizzi Coelho Filho, Pedro Vellosa Schwartzmann, Reinaldo Bulgarelli Bestetti, Ricardo Mourilhe Rocha, Sabrina Bernadez Pereira, Salvador Rassi, Sandrigo Mangini, Silvia Marinho Martins, Silvia Moreira Ayub Ferreira, Victor Sarli Issa.
